# Identification of technology-based models and efficacy of digital-based pain facial expression assessment tools among children: a systematic review

**DOI:** 10.1186/s12912-025-03451-9

**Published:** 2025-07-11

**Authors:** Gusgus Ghraha Ramdhanie, Dessie Wanda, Nur Agustini, Tomy Abuzairi

**Affiliations:** 1 Department of Pediatric and Fundamental Nursing, Faculty of Nursing, Universitas Padjadjaran, Bandung, West Java Indonesia; 2 Doctoral Nursing Program, Faculty of Nursing, Universitas Indonesia, Depok, West Java Indonesia; 3 Department of Pediatric Nursing, Faculty of Nursing, Universitas Indonesia, Depok, West Java Indonesia; 4 Department of Electrical Engineering, Faculty of Engineering, Universitas Indonesia, Depok, West Java Indonesia

**Keywords:** Children, Digital health, Facial expressions, Pain assessment, Technology

## Abstract

**Background:**

Pain management in children remains a significant challenge due to the lack of appropriate assessment methods. Facial expression-based instruments are widely used as facial expressions serve as a key nonverbal indicator of pain. However, conventional paper-based tools have limitations, including subjective interpretation, observer bias, and low accuracy. To address these challenges, digital technology-based facial recognition systems have emerged as a more objective and reliable alternative. This study aims to identify technology-based models and evaluate the efficacy of digital pain facial expression assessment tools for children. These technology-driven approaches aim to provide more objective and consistent solutions than conventional methods.

**Purpose:**

This study aims to identify the technology-based models and efficacy of digital-based pain facial expression assessment instruments in children with a systematic review approach.

**Methods:**

This systematic review follows the Preferred Reporting Items for Systematic Reviews and Meta-Analyses (PRISMA). The article search used five databases: PubMed, EBSCOhost, ScienceDirect, Scopus, and Google Scholar. The study questions used the PCC (Population, Concept, and Context) research framework guidelines. Children with pain as a population, assessment of pain facial expressions as a concept, and technological efficacy as a context. The inclusion criteria for this study were articles published from 2015 to 2024, full-text and free-text articles, and studies that focused on assessing facial expressions of pain in children. Studies were excluded if the article was not in English, and the research design was a literature review type. Study quality was assessed using the Critical Appraisal Checklist Tools from the Joanna Briggs Institute (JBI).

**Results:**

We found 18 studies that described the technology model for assessing facial expressions of pain using computers and mobile applications through video and image recordings. Overall, this suggests that the model used to assess facial expressions of pain is more effective than conventional or paper-based pain assessments. The developed technology model has many advantages, including good performance, high accuracy, an excellent program, validity, reliability, high sensitivity, specificity, and more sensitive.

**Conclusion:**

The findings of this study demonstrate that technology-based models for facial expression pain assessment provide a more objective, accurate, and efficient alternative to conventional methods. These digital tools, including computer and mobile applications, offer real-time analysis, reduce observer bias, and enhance consistency in pain evaluation. Their accessibility, convenience, and automation further strengthen their potential to revolutionize pediatric pain assessment, addressing the limitations of traditional paper-based approaches. Future research should focus on refining these models to improve accuracy across diverse pediatric populations.

## Introduction

Pediatric patients frequently undergo medical interventions during hospitalization that may induce pain [1]. Experiencing pain throughout hospitalization in children can eventuate in stress and psychological/emotional perturbations [2, 3]. Additionally, pain endured by pediatric populations influences various domains, including impaired sleep, physical alterations, challenges interacting, and disrupted engagement in routine activities [4].

Nurses are pivotal in determining suitable pain evaluation approaches for pediatric populations. A fundamental step nurses can undertake in pain management involves conducting comprehensive pain appraisals to characterize the intensity of suffering the child perceives [5]. Effectively managing pain significantly reduces pediatric issues; thus, rigorous pain evaluation is paramount [6]. Appraising pain furnishes the basis for diagnosis, treatment selection, and management assessment for pediatric patients. Conversely, children face challenges verbally depicting and communicating pain [7]. Therefore, incorporating diversified methodologies for evaluating pain in children is necessary, including discernible behaviours to monitor and self-reports of experienced pain.

Pain assessment is the basis for diagnosis, treatment selection, and management assessment for pediatric patients. Pain assessment methods can be categorized into four main approaches: physiological, observational, self-report, and mixed methods [8–10]. Observational pain assessment methods include FLACC (Face, Legs, Activity, Cry, Consolability), CHEOPS (Children’s Hospital of Eastern Ontario Pain Scale), CRIES (Crying, Requires Oxygen, Increased Vital Signs, Expression, Sleeplessness), NIPS (Neonatal Infant Pain Scale), and N-PASS (Neonatal Pain, Agitation, and Sedation Scale), while self-report pain assessments include FACES, OUCHER, and Wong-Baker’s FACES [11–13]. Observational pain assessment methods do not require cognitive abilities from the child and are suitable for children who are unable to verbalize their pain. Pain self-report instruments such as (FACES, OUCHER, Wong-Baker’s FACES) require the child to select one face that reflects the intensity of pain so that they can be used for children as young as four years [10, 14].

Pain assessment in children is still a global problem. One of the problems in pain assessment is the need for appropriate assessment methods for children. Although many manual or paper-based pain assessment instruments can be used, they are often not utilized due to their high workload, long usage time, and simplicity [6, 15–17]. On the other hand, potential digital health technologies for clinical data collection in pain evaluation have resulted in more efficient data than paper-based approaches [18], including assessing pain levels in children [19]. Therefore, the potential of technology drives the development of digital-based pain assessment instruments [6].

Facial expression is an important part of the assessment. Facial expression is one of the most reliable non-verbal indicators for assessing pain, especially in patients who cannot communicate verbally, such as children, patients with cognitive impairment, or those who are unconscious [20, 21]. Facial expressions in children can reflect the degree of pain [22]. Children often express pain through facial expressions to provide essential clues for medical personnel in assessing pediatric pain [20]. This is why developing several pain assessment instruments through facial expression recognition will help improve objective pain assessment accuracy, rather than conventional or paper-based pain assessment tools.

As information technology in healthcare develops, facial detection technology can effectively identify and analyze various subtle features of facial expressions with pain [22]. Pain detection through facial expressions has provided a foundation for objective pain evaluation. Recognition of facial expressions of pain with facial detection technology enables more accurate and objective pain measurement [23]. With detailed visual analysis, this system can assist healthcare workers in understanding and managing patients’ pain more effectively based on detected facial expression signs.

The development of face detection technology in recognizing faces with pain still needs to be explored to provide an objective measure of pain perception. However, a multimodal approach is required to improve the accuracy of face detection technology in recognizing pediatric pain, so developing these systems requires careful validation and testing. Using a systematic review, this study aimed to identify technology-based models and the efficacy of digital-based pain facial expression assessment instruments in children.

## Materials and methods

### Study design

This study used a systematic review approach [24–26]. This method is related to the purpose of the study, which is to explore comprehensively and in-depth to obtain new integrated conclusions by combining previous study data relevant to the specified review question [26, 27]. The study used the Preferred Reporting Items for Systematic Reviews and Meta-Analyses (PRISMA) [26]. Several stages of the study were carried out, including clarifying the aims and objectives, finding relevant studies, collecting data, assessing study quality, and synthesizing and interpreting findings [24].

### Search strategy

Articles were searched through five databases, namely PubMed, EBSCOhost, ScienceDirect, Scopus, and grey literature (Google Scholar). The study questions used the PCC (Population, Concept, and Context) research framework guidelines: children with pain as population, pain expression assessment as concept, and technology efficacy as context. The keyword search used the Term Medical Subject Heading (Mesh) and Boolean operators AND and OR. English keywords were “pediatric” OR “children” OR “kids” OR “adolescents” OR “baby” OR “infant” OR “youth” OR “child” OR “teenager” OR “childhood” AND “pain” AND “facial recognition” OR “facial expression” OR “face detection” OR “face recognition” OR “face recognition technology” OR “face processing” OR “digital face recognition” OR “artificial intelligence” AND “efficacy” OR “evaluation” OR “assessment” OR “accuracy” OR “accurate” OR “validity” OR “reliability” OR “effectiveness”.

### Eligibility criteria

The inclusion criteria for this study were articles published between 2015 and 2024, full-text and free-text articles and studies that focused on assessing facial expressions of pain in children. Studies were excluded if the articles were not in English, and the research design was a literature review type. Three authors independently screened the articles to ensure accuracy and reduce bias.

### Critical appraisal

Study quality was assessed using the critical appraisal checklist tools from the Joanna Briggs Institute (JBI) [28]. Each tool contained questions categorized into four response options: yes, no, unclear, and not applicable. A score of 0 was assigned for “No” and 1 for “Yes.” The evaluation of experimental studies using the JBI checklist for quasi-experimental studies was consistent, comprising nine questions, with a total quality score ranging from 0 to 9. The checklist tool for randomized controlled trials (RCTS) consisted of thirteen questions, with a total quality score ranging from 0 to 13. The checklist tool for feasibility and usability studies, mixed-method studies, and validity and reliability studies followed the diagnostic test accuracy checklist, which included ten questions, with a total quality score ranging from 0 to 10. Based on the cohort studies checklist, the checklist tool for observational studies and pilot exploratory studies comprised eleven questions, with a total quality score ranging from 0 to 11. (See Table 1).

**Table 1:** Critical appraisal tool

Study	Study design	Checklist tools	JBI critical appraisal
Karan Sikka et al. (2015)	Experimental study	Checklist for Quasi-Experimental Studies	7/9 (77,77%)
Chang et al. (2015)	Validity and reliability study	Checklist for Diagnostic test accuracy studies	9/10 (90%)
T. Sun et al. (2015)	RCT	Checklist for RCTs	9/13 (69,23%)
Hadden et al. (2015)	Validity study	Checklist for Diagnostic test accuracy studies	8/10 (80%)
LaFond et al. (2015)	Mixed-methods study	Checklist for Diagnostic test accuracy studies	6/10 (60%)
Zhi et al. (2018)	Experimental study	Checklist for Quasi-Experimental Studies	7/9 (77,77%)
Sakulchit et al. (2019)	Pilot exploratory study	Checklist for Cohort studies	7/11 (63,63%)
Y. Sun et al. (2019)	Experimental study	Checklist for Quasi-Experimental Studies	7/9 (77,77%)
Xu, Craig et al. (2019)	Experimental study	Checklist for Quasi-Experimental Studies	4/9 (44,44%)
Kappesser et al. (2019)	RCT	Checklist for RCTs	9/13 (69,23%)
Xu Susam et al. (2019)	Experimental study	Checklist for Quasi-Experimental Studies	5/9 (55,55%)
Martínez et al. (2020)	Experimental study	Checklist for Quasi-Experimental Studies	7/9 (77,77%)
Hoti et al. (2021)	Feasibility study	Checklist for Diagnostic test accuracy studies	10/10 (100%)
Carlini et al. (2021)	Experimental study	Checklist for Quasi-Experimental Studies	4/9 (44,44%)
Susam et al. (2022)	Experimental study	Checklist for Quasi-Experimental Studies	5/9 (55,55%)
Aydın & Özyazıcıoğlu (2023)	Observational study	Checklist for Cohort studies	7/11 (63,63%)
Hughes et al. (2023)	Feasibility and Usability Evaluation Study	Checklist for Diagnostic test accuracy studies	9/10 (90%)
Talaat et al. (2024)	Experimental study	Checklist for Quasi-Experimental Studies	6/9 (66,66%)

Abbreviations: Randomized control trial, (RCT)

### Data extraction and analysis

Data were extracted using Microsoft Excel (Microsoft Corp, New York, USA) and presented as a tabular matrix. The component groups analyzed in this study are (1) Study characteristics, author name, country, study design, disease type or type, sample size, age, population group, pain type, developed system, and detection model; (2) Development of pain facial expression assessment technology systems from selected studies, including author’s name, developed system, collection procedure, duration, machine learning model (analyzer), detection time, instrument, software, facial detection algorithm, facial detection features, and pain indicators; (3) Summary of results findings from selected studies, including author, type/type of pain, developed system, device type, and results. Data extraction was conducted independently by three authors to minimize bias and improve the reliability of the process. Where discrepancies existed, the authors engaged in discussions to reach consensus. Then, the researcher analyses, summarises, and compiles a results report.

## Result

### Description of study selection

**Fig. 1:**
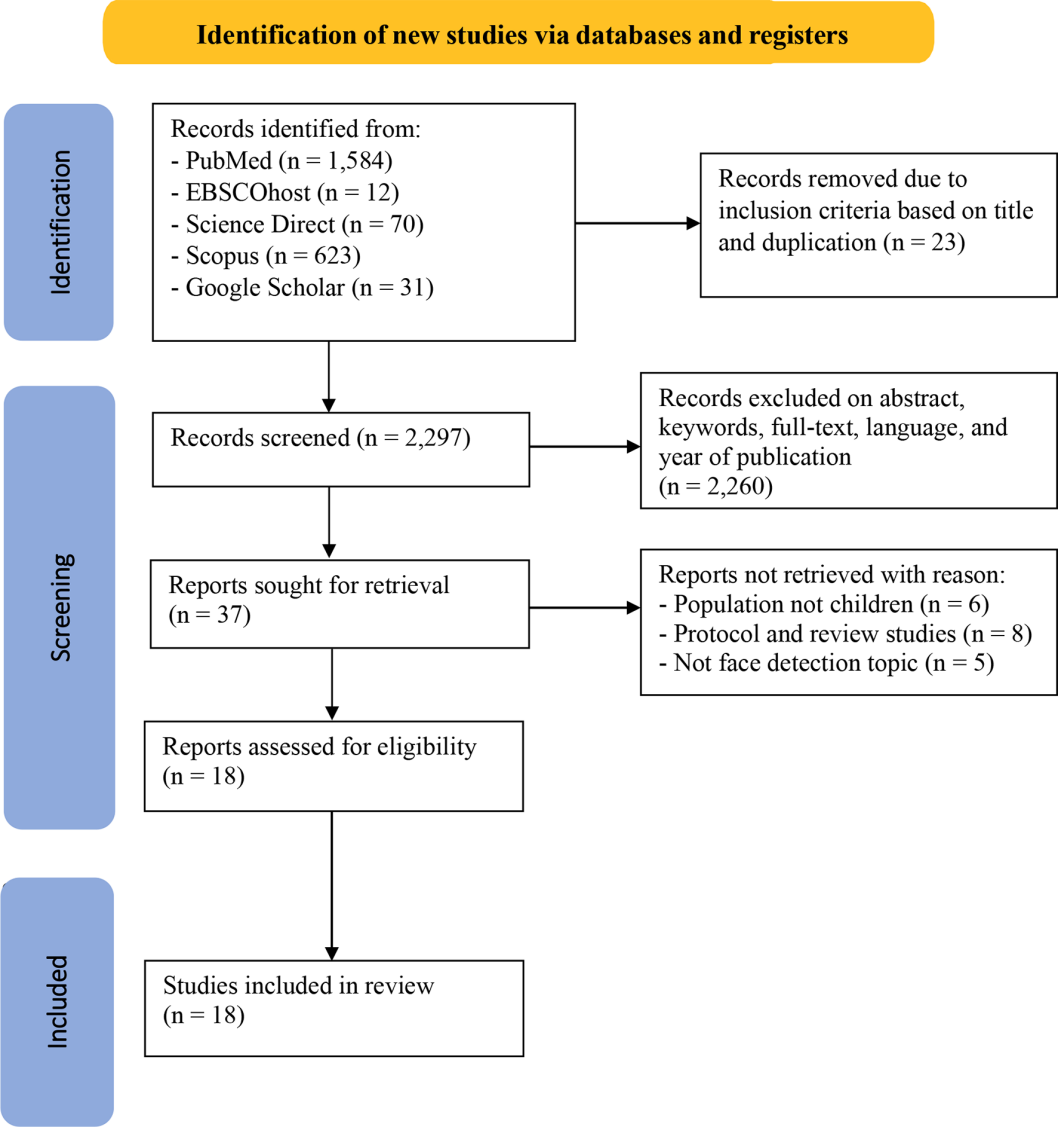
PRISMA flow diagram

The number of articles obtained from the database used was 7,421 articles. Then, screening articles based on title and duplication obtained 2,297 articles. Furthermore, researchers conducted screening by excluding articles based on abstracts, full text, language, and year of publication, and 37 articles were obtained. After the next screening, 19 reports were not taken because the population was not children, the type of literature review study was not a topic related to face detection, so 18 articles were analyzed in this study (Fig. 1). Then, the articles were analyzed using the JBI Critical Appraisal Tool assessment (Table 1).

### Characteristics of study

**Fig. 2:**
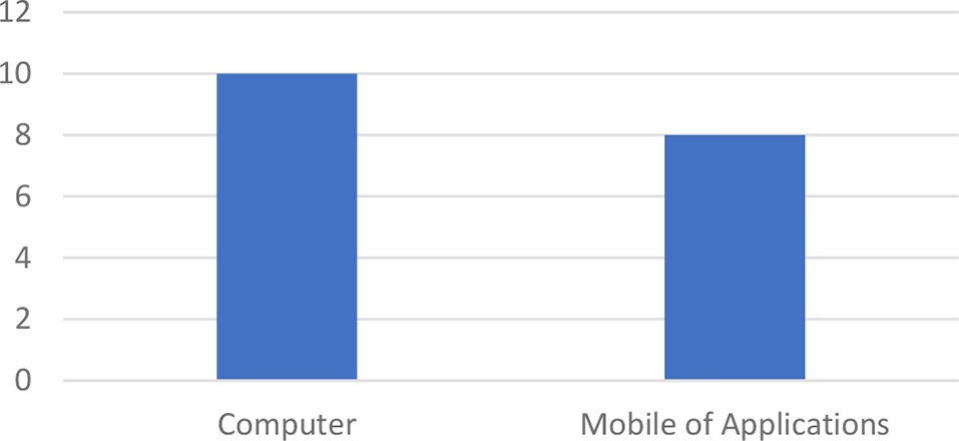
Types of devices used in digital-based tools for facial pain expression assessment

**Fig. 3:**
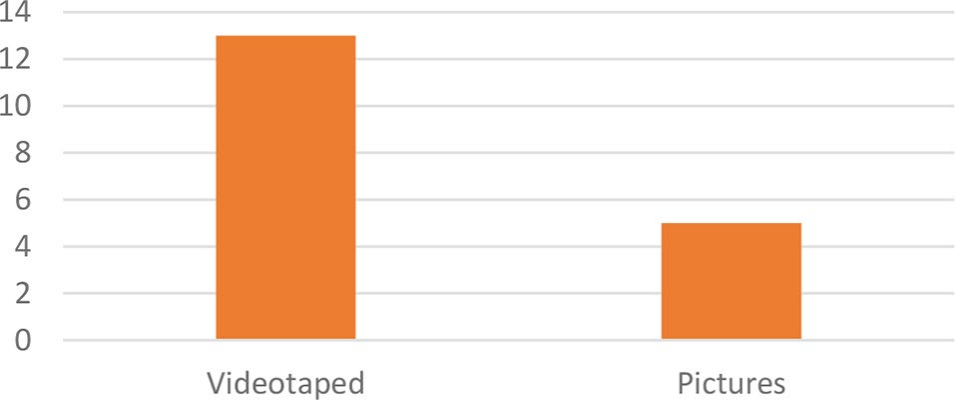
Types of recording used in digital-based tools for facial pain expression assessment

The studies included observational studies (*n* = 2), experimental studies (*n* = 10), feasibility and usability studies (*n* = 2), RCTS (*n* = 2), exploratory pilot studies (*n* = 1), and mixed method studies (*n* = 1). The total sample of study participants was 864 children with various conditions undergoing invasive and postoperative procedures with an age range of 18 h to 18 years. In this study, there were two types of pain in children, including 510 children experiencing postoperative pain (*n* = 9) and 366 children experiencing pain due to invasive procedures (*n* = 8); two studies did not describe the characteristics of children’s pain. Of the 18 studies analyzed, 2 included white children, one included white and black children, and 1 included white and Asian children. The studies were conducted in various countries, including Turkey (*n* = 1), Australia (*n* = 2), Netherlands (*n* = 1), Canada (*n* = 4), Spain (*n* = 1), China (*n* = 1), United States (*n* = 4), Germany (*n* = 1), African-America (*n* = 1), Brazil (*n* = 1), and Egypt (*n* = 1). By country region, studies were conducted in Asia (*n* = 3), Australia (*n* = 2), Europe (*n* = 3), and America (*n* = 10) (See Table 1). The analyzed studies demonstrate the detection model used to assess facial expressions of pain in children. The devices used in the studies were computer devices (*n* = 10) and mobile applications (*n* = 8) (See Fig. 2), while the types of facial recordings were in the form of videos taped (*n* = 13) and figures (*n* = 5) (See Fig. 3).

### Development of a pain facial expression assessment technology system

The technology developed, namely the assessment of facial expressions of pain using computer devices and mobile applications with video and image recording types (See Table 2). Some technologies use facial coding using The Child Facial Coding System (CFCS), Facial Action Coding System (FACS) and Neonatal Facial Coding System (NFCS) or The Neonatal Facial Coding System-Revised (NFCS-R) in the analysis. The facial recordings used for analysis by the machine vary from less than 60 s to 10 min. Meanwhile, the time to generate results ranges from less than one second to 15 min, with the most commonly used duration to generate face detection results being 10 s, especially for CFCS and FACS. Whereas NFCS only requires less than 60 s of detection time. The Numerical Rating Scale (NRS) is the most widely used of the various pain assessment instruments. Most face detection algorithms use automatic Facial Action Unit (AUS) coding, and the developed features can detect the child’s facial area, including specific points on the facial area. The pain indicators developed are facial expressions such as smiles and grimaces (See Table 3).

**Table 2:** Characteristic of included studies (*n* = 18)

Ref	Country	Study design	Population (Type of disease)	Sample (*N*)	Age	Population group	Type of pain	Developed system	Detection model	
									Type of device	Type of recording face
Karan Sikka et al. (2015)	USA	Experimental study	Appendicitis	50	5–18 y	NA	Postoperative	CVML model	Computer	Videotaped
Chang et al. (2015)	Canada	Validity and reliability study	Children with developmental disabilities: myringotomies, tonsillectomies, adenoidectomie, cyst, inguinal hernia, tympanostomy, microlaryngoscopy, laryngobronchoscopy, arch bars, submandibular duct calculus, release of tendons in the hand, electrocautery, and oral lesion	44	1 to 6 y	NA	Postoperative	Multidimensional pediatric observational pain scales	Computer	Videotaped
T. Sun et al. (2015)	Canada	RCT	ASA I–III	62	4–18 y	NA	Postoperative	Panda	Mobile application	Pictures
Hadden et al. (2015)	Canada	Validity study	Cerebral Palsy	85	2–18 y	NA	Physiotherapy (Invasive)	CFCS	Computer	Videotaped
LaFond et al. (2015)	African-American	Mixed-methods study	Critically Ill: sickle cell vaso-occlusive crisis or abdominal disease	4	9 to 11 y	White (82.5%)	Postoperative	VH Vignette	Computer	Pictures
Zhi et al. (2018)	China	Experimental study	NA	12	8 to 10 month	White (10) and Black (2)	NA	Dynamic Hybrid Descriptions	Computer	Videotaped
Sakulchit et al. (2019)	Canada	Pilot exploratory study	NA	77	One month and six years	White (54%) Asian (19%) Others (27%)	Needle penetration Injection (Invasive)	Digital Face Recognition Technology	Mobile application	Pictures
Y. Sun et al. (2019)	Netherlands	Experimental study	NA	24	Two days and 13 months	NA	IV line, venipuncture, vaccination, postoperative (Postoperative and Invasive)	NA	Computer	Videotaped
(Xu, Craig, et al., 2019)	USA	Experimental study	Appendicitis	143	12 y	NA	Postoperative	Human-Assisted Transfer Learning	Mobile application	Videotaped
Kappesser et al. (2019)	Germany	RCT	NA	44	One day	NA	Venipuncture or placing a peripheral venous catheter (Invasive)	Children and Infant’s Postoperative Pain Scale and the Neonatal Facial Coding System– Revised	Computer	Videotaped
Xu Susam et al. (2019)	USA	Experimental study	Appendicitis	42	13	NA	Postoperative	Electrodermal Activity	Computer	Videotaped
Martínez et al. (2020)	Spain	Experimental study	NA	26	18 h and three days	NA	The heel test (Invasive)	Application of Texture Descriptors	Mobile application	Pictures
Hoti et al. (2021)	Australia	Feasibility study	NA	40	2–6 month	NA	Immunizations (Invasive)	PainChek: Point-of-care mobile	Mobile application	Videotaped
Carlini et al. (2021)	Brazil	Experimental study	Healthy neonates	30	11 − 7 days	NA	Venipuncture, capillary, or intramuscular injection (Invasive)	The UNIFESP	Mobile Application	Videotaped
Susam et al. (2022)	USA	Experimental study	Appendicitis	58	5–17 y	NA	Postoperative	Electrodermal Activity and Video Data Fusion	Computer	Videotaped
Aydın & Özyazıcıoğlu (2023)	Turkey	Observational study	Gastrointestinal system, Urinary system, Thoracic, Oncological, and Otorhinolaryngology	83	7–18 y	NA	Postoperative	Computer-assisted facial expression analysis	Computer	Videotaped
Hughes et al. (2023)	Australia	Feasibility and Usability Evaluation Study	NA	40	2–6 month	White (60%)	Immunizations (Invasive)	PainChek	Mobile application	Videotaped
Talaat et al. (2024)	Egypt	Experimental study	Autism	NA	NA	NA	NA	Real-time facial emotion	Mobile application	Pictures

Abbreviations: Computer vision (CV) and machine-learning (ML), (CVML); Computer vision, (CV); Intravenous, (IV); Machine-learning, (ML); Not Applicable, (NA); Randomized controlled trial, (RCT); The Child Facial Coding System, (CFCS); Virtual Human, (VH); years, (y)

**Table 3:** Development of a pain facial expression assessment technology system of included studies (*n* = 18)

Ref	Developed system	Collecting procedures	Duration	Machine learning model analyzer	Time to generating	Instrument	Software	Algoritma detection facial	Features detection facial	Pain indicator
Karan Sikka et al. (2015)	CVML model	Facial activity was recorded for five minutes as a measure of ongoing pain. Then, video recordings of facial activity were collected as representative transient pain samples when manual pressure was applied to the surgical site at two periods of 10 s each.	10 s	CERT^29^	NA	NRS	Emotient Analytics (San Diego, CA)	AUs	AU4: Brow lower, AU6: Cheek raiser (orbit tighten), AU7: Lid tightener, AU9: Nose wrinkler, AU10: Upper lip raiser, AU12: Lip corner puller, AU25: Lips part, AU26: Jaw drop, AU27: Mouth stretch, AU43: Eye closure	Facial expressions
Chang et al. (2015)	Multidimensional pediatric observational pain scales	The average initial duration of video recording was 35 min, ranging from 2 to 62 min. Then, each real-time video recording was edited to provide two 10-second intervals that might indicate pain	10 s	CFCS	10 s	The CFCS	NA	NA	brow lower, eye squeeze, squint, blink, flared nostril, nose wrinkle, nasolabial furrow, cheek raise, open lips, upper lip raise, lip corner pull, horizontal mouth stretch, vertical mouth stretch, blink, flared nostril, and open lips.	Facial expressions
T. Sun et al. (2015)	Panda	The analyzed face image measures 1.1 cm by 0.8 cm when not selected and 2.2 cm by 1.6 cm when selected, compared to 3.3 cm by 2.4 cm for the original paper tool	NA	FPS-R	10 min	FPS-R	iPod TouchTM and REDCap	NA	Face horizontally: neutral, furthest left, and furthest right	Facial expressions
Hadden et al. (2015)	CFCS	Passive joint stretching involves the physiotherapist moving the hamstrings or heels. Children were videotaped for three-time segments: (1) 10 min before the pain condition, (2) during the pain condition (passive joint stretching), and (3) 10 min after the pain condition (recovery)	30 s	CFCS	10 s	NRS and CGCS	NA	AUs	Brow lower, Squint, Eye squeeze, Blink, Flared nostril, Nose wrinkler, Nosolabial furrow, Cheekraiser, Open lips, Upper lip raise, Lip corner puller, Vertical mouth, Horizontal mouth	Facial expressions
LaFond et al. (2015)(LaFond et al., 2015)	VH Vignette	Photos were used to form base head models for the four virtual humans	NA	FACS	NA	PBPQ	NA	VH vignettes	Neutral expression, Smile, and grimace	Facial expressions
Zhi et al. (2018)	Dynamic Hybrid Descriptions	First, several distance parameters were extracted from each video to capture pain-related facial changes. Then, the static parameters consist of a series of temporal signals from several characterization descriptors to obtain the temporal features of the geometric representation of the face.	NA	NFCS	less than a second	NIPS	NA	SVM	brow bulge, nasolabial furrow, eye squeeze, chin quiver, open lips, lip purse, horizontal mouth stretch, vertical mouth stretch, taut tongue, and tongue protrusion	Facial expressions
Sakulchit et al. (2019)	Digital Face Recognition Technology	Pictures were taken in 3 parts, namely before, during, and after needle penetration into the skin	NA	Emotion API	A second	FLACC	API software	NA	Face, lets, activity, cry, and consolability	Facial expressions
Y. Sun et al. (2019)	NA	Faces were recorded when the child was experiencing moments of stress and pain. Video segments vary in length from less than 1 min to several minutes.	less than 60 s	CNN	5 s	NA	DenseNet	CNN	Brow bulge, Nasolabial furrow, Eye squeeze, and Mouth stretch	Facial expressions
Xu, Craig et al. (2019)(Xu, Craig, et al., 2019)	Human-Assisted Transfer Learning	The video was collected during three visits: first, within 24 h after an appendectomy; second, within calendar days after the first visit; and third, at the 25-day postoperative follow-up visit when the pain is thought to have completely subsided. At each visit, two 10-second videos of the face are recorded while manual pressure is applied to the surgical site for 10 s (equivalent to a clinical examination)	10 s	FACS	10 s	NRS	iMotions	AUs	AU1: Inner brow raiser, AU2: Outer brow raiser, AU4: Brow lowerer, AU5: Upper lid raiser, AU6: Cheek raiser and Lid compressor, AU7: Lid tightener, AU9: Nose wrinkler, AU10: Upper lip raiser, AU12: Lip comer puller, AU14: Dimpler, AU15: Lip corner depressor, AU17: Chin raiser, AU18: Lip pucker, AU20: Lip strecher, AU23: Lip tightener, AU24: Lip pressor, AU25: Lips part, AU26: jaw drop, AU28: Lip suck, AU43: Eyes closed	Facial expressions
Kappesser et al. (2019)	Children and Infant’s Postoperative Pain Scale and the Neonatal Facial Coding System– Revised	The video was taken in two different situations, namely when the situation was painful and when the situation was potentially stressful but not painful	10 s	NFCS -R	15 s	NRS and NFCS - R	GiViPAIN	AUs	brow bulge, eye squeeze, nasolabial furrow, horizontal mouth stretch, and taut tongue	Facial expressions
Xu Susam et al. (2019)	Electrodermal Activity	The video was collected during three visits: first, within 24 h after an appendectomy; second, within calendar days after the first visit; and third, at the 42-day postoperative follow-up visit when the pain is thought to have completely subsided. At each visit, two 10-second videos of the face are recorded while manual pressure is applied to the surgical site for 10 s (equivalent to a clinical examination)	10 s	FACS	10 s	NRS	iMotions software	AUs	AU1: Inner brow raiser, AU2: Outer brow raiser, AU4: Brow lowerer, AU5: Upper lid raiser, AU6: Cheek raiser and Lid compressor, AU7: Lid tightener, AU9: Nose wrinkler, AU10: Upper lip raiser, AU12: Lip comer puller, AU14: Dimpler, AU15: Lip corner depressor, AU17: Chin raiser, AU18: Lip pucker, AU20: Lip strecher, AU23: Lip tightener, AU24: Lip pressor, AU25: Lips part, AU26: jaw drop. AU28: Lip suck, AU43: Eyes closed	Facial expressions
Martínez et al. (2020)	Application of Texture Descriptors	A database of images of neonates who have been exposed to pain was used in the heel test. The mobile device and/or wearable system used for the baby monitor will continuously analyze the images it captures. In addition, parents or medical personnel will wear a bracelet to monitor pain	NA	NFCS	less than a second	NFCS	MATLAB ^c^ R2017	SVM	Forehead protusion, Contraction of eyelidsHorizontal stretch of the mouth, Tense tongue, Nasonabial groove	Facial expressions
Hoti et al. (2021)	PainChek: Point-of-care mobile	10-second video recordings were obtained from baseline, during vaccine preparation, during vaccination, and recovery	150 s	AI: FACS	10 s	NFCS-R and ObsVAS	iPad Mini-4 (Cupertino, CA, USA, IOS version 13.6.1).	AUs	AU4: brow lowering, AU9: wrinkling of nose, AU15: lip corner depressor, AU20: horizontal mouth stretch, AU25: parting lips, AU43: eye closure	Facial expressions
Carlini et al. (2021)	The UNIFESP	A 10-minute video was recorded before, during, and after the painful procedure. Images are extracted every three seconds after the video is recorded.	10 min	the Face Detection API from the Firebase’s ML Kit	three seconds	NA	Android Studio IDE	AI	Five facial landmarks (NA)	Facial expressions
Susam et al. (2022)	Electrodermal Activity and Video Data Fusion	At each visit, physiological (EDA) and behavioral (video) reactions were recorded during 10-second intervals of manual abdominal pressure applied near the surgical incision site.	30 s	SVM	30 s	NRS	iMotions	AUs	AU1:Inner brow raiser, AU2: Outer brow raiser, AU4: Brow lowerer, AU5: Upper lid raiser, AU6: Cheek raiser and Lid compressor, AU7: Lid tightener, AU9: Nose wrinkler, AU10: Upper lip raiser, AU12: Lip comer puller, AU14: Dimpler, AU15: Lip corner depressor, AU17: Chin raiser, AU18: Lip pucker, AU20: Lip strecher, AU23; Lip tightener, AU24: Lip pressor, AU25: Lips part, AU26: jaw drop, AU28: Lip suck, AU43: Eyes closed	Facial expressions
Aydın & Özyazıcıoğlu (2023)	Computer-assisted facial expression analysis	Pain assessment was performed at two follow-ups after surgery (within the first 12 h and between the 24th and 36th hours)	60 s	FACS	NA	WBS, VAS and FACS	OpenFace and Python	AUs	AU4: brow lowerer, AU6: cheek raiser, AU7: lid tightener, AU9: nose wrinkler, AU10: upper lip raiser, AU12: lip corner puller, AU17: chin raiser, AU20: lip stretched, AU25: lips part, AU26: jaw drop, AU45: blink	Facial expressions
Hughes et al. (2023)	PainChek	The video was recorded before, during, and after immunization	10 s	NFCS-R	5 min	PainChek Infant, NFCS-R, and ObsVAS	iOS Version 14.0 (Apple Inc)	AUs	Brow lowering, Wrinking of nose, Lip corner depressor, Horizontal mouth stretch, Perting lips, Closing eyes	Facial expressions
Talaat et al. (2024)	Real-time facial emotion	The picture was taken using a gadget. Priority high if the captured image was taken at a remote location from its parent location. The priority is low if the captured images were taken at the same location as	NA	EDA	15 min	NA	Cloud and IoT	DCNN and autoencoder	Face area	Facial expressions

Abbreviations: Application Programing Interface, (API); Artificial Intelligence, (AI); Automatically detect Facial Action Units, (AUs); Convolutional Neural Network, (CNN); Face, lets, activity, cry, and consolability scale, (FLACC); Facial Action Coding System, (FACS); Minutes, (min); Neonatal Facial Coding System, (NFCS); Neonatal Infant Pain Scale, (NIPS); Numerical Rating Scale, (NRS); Second, (s); Support Vector Machine, (SVM); The Child Facial Coding System, (CFCS); The Computer Expression Recognition Toolbox, (CERT)^29^; The Emotion Detection Assistant, (EDA); The Faces Pain Scale-Revised, (FPS-R); The Neonatal Facial Coding System– Revised, (NFCS -R); The Neonatal Facial Coding System-Revised, (NFCS-R); The Observer administered Visual Analogue Scale, (ObsVAS); The Pain Beliefs and Practices Questionnaire, (PBPQ); Visual Analog Scale, (VAS); Wong Baker Faces Pain Scale, (WBS)

### Summary findings

Overall, the model used in assessing facial expressions of pain is considered adequate as a pain detector in children, where the developed technology model has many advantages, including having good performance, showing high accuracy, excellent program, valid, reliable, greater sensitivity and specificity, and the automation code used is more sensitive [12, 13, 19, 22, 29–41].

This study highlights that computer technology and mobile applications have enormous potential as instruments to assess facial expressions of pain in children, especially postoperative pain and invasive procedures (See Table 4). Another finding of this study is that identifying facial expressions of pain in children by analyzing the characteristics of specific population groups still needs to be improved.

**Table 4:** Summary findings of results from included studies (*n* = 18)

Ref	Type of pain	Developed system	Type of device	Result
Karan Sikka et al. (2015)	Postoperative	CVML model	Computer	The CVML pain assessment model of automated facial expression measurements demonstrated good to excellent accuracy in binary pain classification, strong correlation with patient-reported pain ratings and estimation of pain levels after appendectomy
Chang et al. (2015)	Postoperative	Multidimensional pediatric observational pain scales	Computer	These findings indicate that variations in cues proposed to assess facial expression pain lead to a wide range of scores and may be insensitive to differences in children’s pain intensity.
T. Sun et al. (2015)	Postoperative	Panda	Mobile application	Assessment of facial expressions of pain using Panda results correlated strongly with original scores at both time points (Pearson’s *r* > 0.87), with higher average pain scores (up to + 0.47 out of 10). Additionally, Panda represents a slight systematic bias. Participants who prefer Panda over the original tool (81% of FPS-R). The Panda application can be used to assess facial expressions and pain in children
Hadden et al. (2015)	Physiotherapy (Invasive)	CFCS	Computer	The results show that Pediatric Facial Coding System scores during passive joint stretching were significantly correlated with Numerical Rating Scale scores (r ¼ 0.72, *P* < 0.01). CFCS scores were also significantly higher during passive joint stretching than baseline and recovery segments (*P* < 0.001). These findings suggest that PFCS is a valid method for identifying facial expression pain in children with Cerebral Palsy.
LaFond et al. (2015)(LaFond et al., 2015)	Postoperative	VH Vignette	Computer	Facial expression recognition was 98.4%. The pain of smiling children was rated significantly lower than that of children who grimaced in the VH and written vignettes. Pain was rated significantly lower in children who grimaced in VH vignettes than in written vignettes. VH vignette pain ratings correlated strongly with the written version. This method was effective for developing VH vignettes that demonstrated good face validity across participants and convergent validity with written vignettes
Zhi et al. (2018)	NA	Dynamic Hybrid Descriptions	Computer	The results show that the best accuracy of the proposed facial expression pain assessment approach was 95.6%. Additionally, decision fusion does not perform better than feature fusion. The False Negative Rate of decision fusion (6.2%) is much lower compared to feature fusion (25%)
Sakulchit et al. (2019)	Needle penetration Injection (Invasive)	Digital Face Recognition Technology	Mobile application	During needle penetration, SADNESS was significantly correlated (0.887, *P* < 0.05). The maximum correlation for each emotion shows an increase in SADNESS and a decrease in NEUTRAL emotions compared to before needle penetration. During the blood test procedure, young children showed higher SADNESS and lower NEUTRAL emotions, as reported by Emotion API
Y. Sun et al. (2019)	Intravenous (IV) line, venipuncture, vaccination, postoperative (Postoperative and invasive)	NA	Computer	This method provides better results than existing methods based on handcrafted features. Combining individual frame results further improves the AUC from 0.96 to 0.98. The proposed system has great potential for continuous monitoring of discomfort and pain based on facial expression pain
Xu, Craig et al. (2019)(Xu, Craig, et al., 2019)	Postoperative	Human-Assisted Transfer Learning	Mobile application	We found that automatically coded AUs were more sensitive than AUs coded by humans trained in the FACS system for assessing facial expressions of pain.
Kappesser et al. (2019)	Venipuncture or placing a peripheral venous catheter (Invasive)	Children and Infant’s Postoperative Pain Scale and the Neonatal Facial Coding System– Revised	Computer	This study shows that the assessment tools used had relatively high reliability, internal consistency, and convergent validity. Both tools can differentiate between painful and stressful situations well through facial expressions.
Xu Susam et al. (2019)	Postoperative	Electrodermal Activity	Computer	This study shows the results of using a fusion approach to detect pain in children via facial expressions of pain automatically. The developed fusion methodology improved pain assessment compared to approaches using video features, transferred video, or EDA only.
Martínez et al. (2020)	The heel test (Invasive)	Application of Texture Descriptors	Mobile application	The results show that the features used provide a very promising classification accuracy of around 95% for the Infant COPE database, proving the method’s validity for measuring facial expression pain in children.
Hoti et al. (2021)	Immunizations (Invasive)	PainChek: Point-of-care mobile	Mobile application	The mobile application-based PainChek, as a facial expression pain assessment tool, showed significant changes in recorded pain scores from videos (*p* ≤ 0·0006). Infant PainChek pain scores correlated well with NFCS-R and ObsVAS scores (*p* < 0·0001). PainChek Infant also demonstrated good to excellent inter-rater reliability (*p* < 0·001) and high levels of internal consistency (α = 0·82–0·97)
Carlini et al. (2021)	Venipuncture, capillary, or intramuscular injection (Invasive)	The UNIFESP	Mobile Application	Studies show that this method can accurately classify facial expressions of neonatal pain. Results highlight facial regions that are suitable for use in assessing pain scales by facial expressions
Susam et al. (2022)	Postoperative	Electrodermal Activity and Video Data Fusion	Computer	The results show that EDA and facial expression data independently provide greater sensitivity and specificity, but the combination of both to classify clinically significant pain and clinically insignificant pain achieves a substantial improvement in accuracy (90.91%) with sensitivity ( 100%) and specificity (81.82%).
Aydın & Özyazıcıoğlu (2023)	Postoperative	Computer-assisted facial expression analysis	Computer	The methods used in assessing pain in children have good performance in estimating the severity of pain. This method can code facial expressions of facial expression pain in children and reliably measure pain from video recordings
Hughes et al. (2023)	Immunizations (Invasive)	PainChek	Mobile application	This study shows that PainChek Infant Standard and Adaptive demonstrate high accuracy. Accuracy and precision are 0.908 and 0.912, respectively, for Standard and 0.912 and 0.897 for Adaptive mode. PainChek Infant performed well on all aspects of feasibility, including interpretability (defined cut-off scores), ease of administration, turnaround time of only 3 s, and clinical understanding.
Talaat et al. (2024)	NA	Real-time facial emotion	Mobile application	This study shows the potential of facial expression analysis and deep learning algorithms for real-time emotion recognition in autistic children. This system can detect six facial emotions, namely anger, emotion, acuteness, sadness, joy and surprise

Application Programing Interface, (API); Computer vision (CV) and machine-learning (ML), (CVML); Facial Action Coding System, (FACS); Not Applicable, (NA**);** The Area Under the Curve (AUC); The Child Facial Coding System, (CFCS); The Emotion Detection Assistant, (EDA); The Faces Pain Scale-Revised, (FPS-R); Virtual Human, (VH)

## Discussion

### Principal finding

This study identifies the development and validates the efficacy of digital technology-based pain facial assessment instruments in children with a systematic review approach. Many studies have shown that technology-based facial expression assessment models are effective as pain detectors in children with various conditions undergoing invasive and postoperative procedures.

Expressions are associated with pain or pain-related distress, not emotions such as sadness or anger [42]. The first finding in this study shows that technology-based detection of facial expressions of pain has many advantages, including strong performance, high accuracy, validity, reliability, and greater sensitivity and specificity. The results of a previous study showed that Pediatric Pain Facial Coding System scores were significantly correlated with Numerical Rating Scale scores [12]. The meta-analysis revealed that electronic methods were superior to conventional pain assessment methods, including ease, efficiency, and acceptability [43].

The second finding is that technology-based pain facial assessment can be used to detect pain in children with various conditions undergoing invasive and postoperative procedures, with an age range of 18 h to 18 years. Previous studies have shown that technology algorithms can assess pain in children aged 1 to 12 months [35]. Another study confirmed these findings by analyzing facial expressions in 2-, 4-, 6-, and 12-month-old infants during invasive procedures, using PainChek Infant and NFCS-R [42]. The NRS is the most widely used pain assessment instrument.

The third finding of this study is that the assessment of children’s facial expressions of pain involves (1) automated analysis tools—computer-based systems and mobile applications—and (2) data acquisition through video recordings and still images (photographs). Videos are particularly effective in capturing dynamic and geometric facial texture features, enabling the classification of pain and non-pain expressions in infants [31]. Multiple studies have demonstrated that video recordings offer higher accuracy than static images in detecting pain in children [44, 45], and also allow for reviewing multiple segments for comprehensive assessment [42]. Nevertheless, still images also contribute effectively to pain detection. Furthermore, mobile applications allow for easy and efficient pain assessment by analyzing facial images and videos. They offer advantages such as cost-effectiveness and practicality [46].

Another key finding of this review is that few studies consider population-specific characteristics when identifying pain-related facial expressions in children. Evidence suggests that facial expressions of pain may vary across demographic groups, including differences influenced by racial or ethnic backgrounds [47, 48]. Therefore, understanding the racial and cultural context is crucial for accurate interpretation and analysis of facial pain expressions in children [49].

Regarding analytical methods, various machine learning models employ facial coding systems such as CFCS, FACS, NFCS, or NFCS-R, with encoders that rely on Action Units (AUs). Facial recording durations ranged from under 60 s to 10 min, and the time required to detect pain ranged from less than one second to 15 min, with an average detection time of 10 s. These models generally demonstrated strong performance in pediatric pain assessment, including high agreement rates when using NFCS-R and significant results using CFCS scores [12, 35, 38].

Artificial intelligence (AI) algorithms—particularly those based on Action Units (AUS)—have demonstrated strong capabilities in recognizing facial expressions of pain and differentiating them from non-pain-related expressions [50, 51]. These algorithms are sensitive to environmental and contextual differences, enabling more accurate detection of pain signals [29]. AUs identify key facial areas using video recordings, allowing detailed and dynamic facial analysis [52, 53]. Overall, this study highlights the significant potential of AI-integrated technologies to support clinical nursing practice, especially in managing postoperative pain and invasive procedures in children.

Technology-based models that assess pain through facial expressions present a promising alternative for evaluating pain in children with neurodevelopmental disorders (NDDS). Facial coding systems, such as the Child Facial Coding System (CFCS), are objective and valid for measuring pain in children with cerebral palsy [12, 13]. Since children with NDDS often struggle to express their pain verbally, facial recognition technology can help improve the objectivity and accuracy of pain assessments. However, it is important to note that children with NDDS may display atypical or variable facial expressions, which could impact the accuracy of these tools. For example, the facial expressions of individuals with cerebral palsy (CP) can differ due to motor and neurological disorders that affect facial muscle control, making pain expressions less consistent or difficult to interpret [54]. In addition, in children with chronic pain, facial expressions may become less pronounced over time due to adaptation, even though significant pain may still be present. These challenges highlight the importance of refining facial recognition systems to improve their performance in assessing pain across diverse pediatric populations, especially those with neurological and developmental differences.

### Strengths and limitations

This study has several limitations, including article searches limited to four databases and one search engine. This makes it possible that there is still literature that needs to be included from other databases, causing the literature to be incomplete. The article quality assessment was only conducted by three researchers, so the assessment results are potentially biased. In addition, this study did not limit the quality score of the article assessment results to reduce the exclusion of articles analyzed.

### Nursing implications

Pain management in children presents an ongoing challenge in clinical practice, particularly in achieving rapid and accurate assessment. This review underscores the potential of technology-based facial expression analysis as a supportive tool for nurses and other healthcare professionals in identifying pain and planning appropriate interventions. To enhance the effectiveness and efficiency of pain assessment, several key recommendations emerge from this review: the use of standardized facial coding systems such as FACS, CFCS, or NFCS; the development of mobile-based applications utilizing video or image input for real-time analysis; and the application of Action Unit (AU) coding to detect specific facial landmarks indicative of pain. These strategies can be a foundation for advancing pain assessment methods in pediatric nursing care. However, further evaluation is needed to determine the cost-effectiveness and clinical feasibility of implementing such technologies in diverse healthcare settings.

## Conclusion

Based on the findings of this systematic review, 18 articles were analyzed describing the assessment of facial expressions of pain in children. The current findings indicate that the model used in assessing facial expressions of pain is considered adequate as a pain detector in children. This systematic review highlights that computer technology and mobile applications have great potential as instruments to assess facial expressions of pain in children, especially postoperative pain and during invasive procedures in children and children in specific populations.

The findings in this systematic review can serve as a reference for nurses and other health professionals in developing more effective and efficient facial expression assessment methods in pediatric pain assessment. Future studies to strengthen the current findings, studies with a meta-analysis approach need to be conducted to assess the effectiveness of various factors from the facial expression assessment technology model of pain in children. In addition, future research in developing digital-based pain assessment tools must consider the racial context for analyzing and interpreting facial expressions in pain.

## Data Availability

All data generated or analyzed during this study are included in this published article.

## References

[1] Vejzovic V, Bozic J, Panova G, Babajic M, Bramhagen A-C. Children still experience pain during hospital stay: a cross-sectional study from four countries in Europe. BMC Pediatr. 2020;20(1):39. 10.1186/s12887-020-1937-1. 31996162 10.1186/s12887-020-1937-1 PMC6988252

[2] Rennick JE, et al. Measuring psychological outcomes following pediatric intensive care unit hospitalization: psychometric analysis of the children’s critical illness impact scale. Pediatr Crit Care Med. 2011;12. 10.1097/PCC.0b013e3182191bfa. 10.1097/PCC.0b013e3182191bfa 21499186

[3] Trottier ED, Doré-Bergeron M-J, Chauvin-Kimoff L, Baerg K, Ali S. Managing pain and distress in children undergoing brief diagnostic and therapeutic procedures., *Paediatr Child Health*, vol. 24, no. 8, pp. 509–535, Dec. 2019, 10.1093/pch/pxz026 10.1093/pch/pxz026 PMC6901171 31844394

[4] Haraldstad K, Sørum R, Eide H, Natvig GK, Helseth S. Pain in children and adolescents: prevalence, impact on daily life, and parents’ perception, a school survey., *Scand J Caring Sci*, vol. 25, no. 1, pp. 27–36, Mar. 2011, 10.1111/j.1471-6712.2010.00785.x 10.1111/j.1471-6712.2010.00785.x 20409061

[5] Saleh AM. Nurses’ assessment and management practices of pain among intensive care patients in King Khalid hospital, kharj, Riyadh. Heliyon. Sep. 2023;9(9):e19986. 10.1016/j.heliyon.2023.e19986. 10.1016/j.heliyon.2023.e19986 PMC10559661 37809981

[6] Manworren RCB, Stinson J, Manworren RCB, Stinson J. Pediatric pain measurement, assessment and evaluation. Semin Pediatr Neurol. 2016. 10.1016/j.spen.2016.10.001. 27989326 10.1016/j.spen.2016.10.001 PMC5261830

[7] Potter MN, Howell DR, Dahab KS, Sweeney EA, Albright JC, Provance AJ. Sleep Quality and Quality of Life Among Healthy High School Athletes., *Clin Pediatr (Phila)*, vol. 59, no. 2, pp. 170–177, Feb. 2020, 10.1177/0009922819892050 10.1177/0009922819892050 31808366

[8] Khoirunnisa YN, Novitasari RW. Assessment Nyeri. Tehnik. 2015;42(3):226–32.

[9] Hockenberry MJ, Wilson D. Wong’s essentials of pediatric nursing. St. Louis: Mosby; 2016.

[10] Crellin D, Harrison D, Santamaria N, Babl FE. Comparison of the psychometric properties of the FLACC scale, the MBPS and the observer applied visual analogue scale used to assess procedural pain. J Pain Res. 2021;14:881–92. 10.2147/JPR.S267839. 33833566 10.2147/JPR.S267839 PMC8020135

[11] Trottier ED, Ali S, Doré-Bergeron MJ, Chauvin-Kimoff L. Best practices in pain assessment and management for children, *Paediatrics and Child Health (Canada)*, vol. 27, no. 7, pp. 429–437, Dec. 2022, 10.1093/pch/pxac048 10.1093/pch/pxac048 PMC9732859 36524020

[12] Hadden KL, Lefort S, O’Brien M, Coyte PC, Guerriere DN. Validity of the child facial coding system for the assessment of acute pain in children with cerebral palsy. J Child Neurol. 2015;31(5):1–6. 10.1177/0883073815604228. 10.1177/0883073815604228 26353879

[13] Chang J, Versloot J, Fashler SR, McCrystal KN, Craig KD. Pain assessment in children: validity of facial expression items in observational pain scales. Clin J Pain. 2015;31(3):189–97. 10.1097/AJP.0000000000000103. 24810648 10.1097/AJP.0000000000000103

[14] Yue J-M, Wang Q, Liu B, Zhou L. Postoperative accurate pain assessment of children and artificial intelligence: A medical hypothesis and planned study. World J Clin Cases. Feb. 2024;12(4):681–7. 10.12998/wjcc.v12.i4.681. 10.12998/wjcc.v12.i4.681 PMC10841123 38322690

[15] Gauchan S. Pain assessment in emergency department of teaching hospital in Lalitpur. J Karnali Acad Health Sci. Dec. 2019;2(3):209–13. 10.3126/jkahs.v2i3.26657.

[16] Fernandes AM, De Campos Bsn C, Batalha L, Perdigão A, Msc, Phd EJ. Pain assessment using the adolescent pediatric pain tool: a systematic review. Pain Res Manag, 19, 4, 2014. 10.1155/2014/979416 PMC4158937 24950413

[17] Drendel AL, Kelly BT, Ali S. Pain Assessment for children overcoming challenges and optimizing care, *Pediatr Emerg Care*, vol. 27, no. 8, pp. 773–781, 2011, Accessed: Mar. 02, 2024. [Online]. Available: https://doi//1097/PEC.0b013e31822877f7. 10.1097/PEC.0b013e31822877f7 21822093

[18] Higgins KS, et al. Availability of researcher-led eHealth tools for pain assessment and management: barriers, facilitators, costs, and design. Pain Rep. Sep. 2018;3(7). 10.1097/PR9.0000000000000686. 10.1097/PR9.0000000000000686 PMC6172815 30324177

[19] Sakulchit T, Kuzeljevic B, Goldman RD. Evaluation of Digital Face Recognition Technology for Pain Assessment in Young Children, *Clin J Pain*, vol. 35, no. 1, pp. 18–22, Jan. 2019, 10.1097/AJP.0000000000000659 10.1097/AJP.0000000000000659 30247199

[20] Benzon H, Rathmell JP, Wu CL, Turk D, Argoff CE, Hurley RW. Practical management of pain E-Book. Volume 2. Elsevier Health Sciences; 2022.

[21] Manworren RCB, Stinson J. Pediatric pain measurement, assessment, and evaluation. Semin Pediatr Neurol. Aug. 2016;23(3):189–200. 10.1016/j.spen.2016.10.001. 10.1016/j.spen.2016.10.001 PMC5261830 27989326

[22] Sikka K, et al. Automated assessment of children’s postoperative pain using computer vision. Pediatrics. 2015;136(1):1–8. 10.1542/peds.2015-0029. 26034245 10.1542/peds.2015-0029 PMC4485009

[23] De Sario GD, et al. Using Ai to detect pain through facial expressions: a review. Bioengineering. May 2023;10(5). 10.3390/bioengineering10050548. 10.3390/bioengineering10050548 PMC10215219 37237618

[24] Aromataris E, Pearson A. The systematic review: an overview. Am J Nurs. 2014;114(3):53–8. 10.1097/01.NAJ.0000444496.24228.2c. 24572533 10.1097/01.NAJ.0000444496.24228.2c

[25] Cope DG. Preferred reporting items for systematic reviews and meta-analyses. Oncol Nurs Forum. 2015;42(5):552–4. 10.1188/15.ONF.552-554. 26302284 10.1188/15.ONF.552-554

[26] Page MJ, et al. The PRISMA 2020 statement: an updated guideline for reporting systematic reviews. BMJ. 2021;372:1–11. 10.1136/bmj.n71. 10.1136/bmj.n71 PMC8005924 33782057

[27] Pollock A, Berge E. How to do a systematic review., *Int J Stroke*, vol. 13, no. 2, pp. 138–156, Feb. 2018, 10.1177/1747493017743796 10.1177/1747493017743796 29148960

[28] Barker TH, Stone JC, Sears K, Klugar M, Tufanaru C, Leonardi-Bee J, et al. The revised JBI critical appraisal tool for the assessment of risk of bias for randomized controlled trials. JBI Evidence Synthesis. 2023;21(3):494–506 10.11124/JBIES-22-00430 36727247

[29] Sun T, et al. A smartphone version of the faces pain scale-Revised and the color analog scale for postoperative pain assessment in children. Paediatr Anaesth. 2015;25(12):1264–73. 10.1111/pan.12790. 26507916 10.1111/pan.12790

[30] LaFond CM, et al. Development and validation of a virtual human vignette to compare nurses’ assessment and intervention choices for pain in critically ill children. Simul Healthc. 2015;10(1):14–20. 10.1097/SIH.0000000000000061.Development. 25514587 10.1097/SIH.0000000000000061 PMC4315722

[31] Zhi R, Zamzmi G, Goldgof D, Ashmeade T, Li T, Sun Y. Infants’ pain recognition based on facial expression: dynamic hybrid descriptions. IEICE Trans Inf Syst. 2018;E101D:1860–9. 10.1587/transinf.2017EDP7272.

[32] Sun Y, et al. Detecting discomfort in infants through facial expressions. Physiol Meas. 2019;1–15. 10.1088/1361-6579/ab55b3. Manuscript. 10.1088/1361-6579/ab55b3 31703212

[33] Kappesser J, de Laffolie J, Faas D, Ehrhardt H, Hermann C. Comparison of two neonatal pain assessment tools (Children and infant’s postoperative pain scale and the neonatal facial coding System—Revised) and their relations to clinicians’ intuitive pain estimates. Eur J Pain (United Kingdom). 2019;23(4):708–18. 10.1002/ejp.1338. 10.1002/ejp.1338 30407684

[34] Martínez A, Pujol FA, Mora H. Application of texture descriptors to facial emotion recognition in infants. Appl Sci (Switzerland). 2020;10:1115. 10.3390/app10031115.

[35] Hoti K, Chivers PT, Hughes JD. Assessing procedural pain in infants: a feasibility study evaluating a point-of-care mobile solution based on automated facial analysis. Lancet Digit Health. 2021;3(10):e623–34. 10.1016/S2589-7500(21)00129-1. 34481769 10.1016/S2589-7500(21)00129-1

[36] Carlini LP et al. A Convolutional Neural Network-based Mobile Application to Bedside Neonatal Pain Assessment, in *Proceedings– 2021 34th SIBGRAPI Conference on Graphics, Patterns and Images, SIBGRAPI 2021*, 2021, pp. 394–401. 10.1109/SIBGRAPI54419.2021.00060

[37] Susam B, et al. Automated pain assessment in children using electrodermal activity and video data fusion via machine learning. IEEE Trans Biomed Eng. 2022;69(1):422–31. 10.1109/TBME.2021.3096137. 34242161 10.1109/TBME.2021.3096137

[38] Aydın Aİ, Özyazıcıoğlu N. Assessment of postoperative pain in children with computer assisted facial expression analysis. J Pediatr Nurs. 2023;71:60–5. 10.1016/j.pedn.2023.03.008. 37004311 10.1016/j.pedn.2023.03.008

[39] Hughes JD, Chivers P, Hoti K. The Clinical Suitability of an Artificial Intelligence-Enabled Pain Assessment Tool for Use in Infants: Feasibility and Usability Evaluation Study, *J Med Internet Res*, vol. 25, no. e41992|, pp. 1–14, 2023, 10.2196/41992 10.2196/41992 PMC9972204 36780223

[40] Talaat FM, Ali ZH, Mostafa RR, El-Rashidy N. Real-time facial emotion recognition model based on kernel autoencoder and convolutional neural network for autism children. Soft Comput. 2024. 10.1007/s00500-023-09477-y.

[41] Xu X, Susam BT, Nezamfar H, Diaz D, Craig KD, Goodwin MS, et al. Towards automated pain detection in children using facial and electrodermal activity. CEUR Workshop Proc. 2018;2142:208–11. PMC6352962 30713486

[42] Kohut SA, Riddell RP, Flora DB, Oster H. A longitudinal analysis of the development of infant facial expressions in response to acute pain: immediate and regulatory expressions. Jurnal Sains Dan Seni ITS. 2017;6(1):51–66. 10.1016/j.pain.2012.09.005 23103435

[43] Jibb LA, et al. Electronic data capture versus conventional data collection methods in clinical pain studies: systematic review and meta-analysis. J Med Internet Res. 2020;22(6). 10.2196/16480. 10.2196/16480 PMC7351264 32348259

[44] Zamzami G, Ruiz G, Goldgof D, Kasturi R, Sun Y, Ashmeade T. Pain assessment in infants: towards spotting pain expression based on infants’ facial strain. 2015 11th IEEE Int Conf Workshops Automatic Face Gesture Recognit FG 2015. 2015;2015–January. 10.1109/FG.2015.7284857.

[45] Zamzmi G, Pai CY, Goldgof D, Kasturi R, Ashmeade T, Sun Y. An approach for automated multimodal analysis of infants’ pain. Proc - Int Conf Pattern Recognit. 2016;0:4148–53. 10.1109/ICPR.2016.7900284.

[46] Hasan MK, Ahsan GMT, Ahamed SI, Love R, Salim R. Pain level detection from facial image captured by smartphone. J Inform Process. 2016;24(4):598–608. 10.2197/ipsjjip.24.598.

[47] Nerella S, Cupka J, Ruppert M, Tighe P, Bihorac A, Rashidi P. Pain action unit detection in critically ill patients. Proc– 2021 IEEE 45th Annual Computers Softw Appl Conf COMPSAC 2021. 2021;645–51. 10.1109/COMPSAC51774.2021.00094. 10.1109/compsac51774.2021.00094 PMC8552410 34723289

[48] Wandner LD, Scipio CD, Hirsh AT, Torres CA, Robinson ME. The perception of pain in others: how gender, race, and age influence pain expectations. J Pain. 2012;13(3):220–7. 10.1016/j.jpain.2011.10.014. 22225969 10.1016/j.jpain.2011.10.014 PMC3294006

[49] Hoffman KM, Trawalter S, Axt JR, Oliver MN. Racial bias in pain assessment and treatment recommendations, and false beliefs about biological differences between Blacks and Whites. Proc Natl Acad Sci U S A. 2016;113(16):4296–301. 10.1073/pnas.1516047113. 27044069 10.1073/pnas.1516047113 PMC4843483

[50] Fontaine D, et al. Artificial intelligence to evaluate postoperative pain based on facial expression recognition. Eur J Pain. 2022;26(6):1282–91. 10.1002/ejp.1948. 35352426 10.1002/ejp.1948

[51] Othman E, Werner P, Saxen F, Al-Hamadi A, Gruss S, Walter S. Automatic vs. Human recognition of pain intensity from facial expression on the x-ite pain database. Sensors. 2021;21(9):1–19. 10.3390/s21093273. 10.3390/s21093273 PMC8125973 34068462

[52] Bartlett MS, Littlewort GC, Frank MG, Lee K. Automatic decoding of facial movements reveals deceptive pain expressions. Curr Biol. 2014;24(7):738–43. 10.1016/j.cub.2014.02.009. 24656830 10.1016/j.cub.2014.02.009 PMC4034269

[53] Martinez B, Valstar MF, Jiang B, Pantic M. Automatic analysis of facial actions: A survey. IEEE Trans Affect Comput. 2019;10(3):325–47. 10.1109/TAFFC.2017.2731763.

[54] Sabater-Gárriz Á, Gaya-Morey FX, Buades-Rubio JM, Manresa-Yee C, Montoya P, Riquelme I. Automated facial recognition system using deep learning for pain assessment in adults with cerebral palsy. Digit Health. Jan. 2024;10. 10.1177/20552076241259664. 10.1177/20552076241259664 PMC11155325 38846372

